# Metabolic and ventilatory changes during postural change from the supine position to the reclining position in bedridden older patients

**DOI:** 10.1097/MD.0000000000033250

**Published:** 2023-03-10

**Authors:** Yoji Yamada, Yuji Mitani, Akio Yamamoto, Kazumo Miura, Kanji Yamada, Yukari Oki, Yutaro Oki, Yasumichi Maejima, Yoko Kurumatani, Akira Ishikawa

**Affiliations:** a Department of Rehabilitation, Isawa Kyoritsu Hospital, Fuefuki, Yamanashi, Japan; b Department of Public Health, Kobe University Graduate School of Health Sciences, Kobe, Hyogo, Japan; c Department of Rehabilitation, Kofu Kyoritsu Hospital, Kofu, Yamanashi, Japan; d Department of Cardiology, Kofu Kyoritsu Hospital, Kofu, Yamanashi 406-0035, Japan.

**Keywords:** bedridden patients, older, pneumonia, rehabilitation, sitting position, tidal volume, V̇O_2_

## Abstract

The prevention of pneumonia in bedridden older patients is important, and its recurrence in these patients is a relevant issue. Patients who are bedridden and inactive, and have dysphagia are considered to be at risk for pneumonia. Efforts to reduce the bedridden state and low activity may be necessary to reduce the risk of developing pneumonia in bedridden older patients. This study aimed to clarify the effects of postural change from the supine position to the reclining position on metabolic and ventilatory parameters and on safety in bedridden older patients. Using a breath gas analyzer and other tools, we assessed the following 3 positions: lying on the back (supine), resting in the Fowler position (Fowler), and resting in an 80° recline wheelchair (80°). Measurements were oxygen uptake, carbon dioxide output, gas exchange ratio, tidal volume (V_T_), minute volume, respiratory rate, inspiratory time, expiratory time, total respiratory time, mean inspiratory flow, metabolic equivalents, end-expiratory oxygen, and end-expiratory carbon dioxide as well as various vital signs. The study analysis included 19 bedridden participants. The change in oxygen uptake driven by changing the posture from the supine position to the Fowler position was as small as 10.8 mL/minute. V_T_ significantly increased from the supine position (398.4 ± 111.2 mL) to the Fowler position (426.9 ± 106.8 mL) (*P* *=* .037) and then showed a decreasing trend in the 80° position (416.8 ± 92.5 mL). For bedridden older patients, sitting in a wheelchair is a very low-impact physical activity, similar to that in normal people. The V_T_ of bedridden older patients was maximal in the Fowler position, and the ventilatory volume did not increase with an increasing reclining angle, unlike that in normal people. These findings suggest that appropriate reclining postures in clinical situations can promote an increase in the ventilatory rate in bedridden older patients.

## 1. Introduction

The prevention of pneumonia in bedridden older patients is important, and its recurrence in these patients is a relevant issue. However, rehabilitation for these patients has not yet been established. Pneumonia is the 4th leading cause of deaths in Japan (https://www.mhlw.go.jp/toukei/itiran/eiyaku.html, Accessed September 5, 2022), and 80.1% of patients with pneumonia aged > 70 years are diagnosed with aspiration pneumonia.^[1]^ Low activities of daily living ^[1]^ are reportedly associated with the development of pneumonia.^[2]^ To prevent the development of pneumonia, vaccination and oral care are strongly recommended.^[3]^ Notably, early rehabilitation for patients with pneumonia has been reported to be effective.^[4]^ In contrast, methods of rehabilitation for preventing the onset of pneumonia in older people have not yet been established.^[2]^

Older patients who are bedridden and inactive and have dysphagia are considered to be at risk for pneumonia. Some previous studies have reported bedridden state, weight loss, gait disturbance, dysphagia, and presence of aspiration, impaired swallowing function, dehydration, and dementia as risk factors for aspiration pneumonia.^[5]^ Recently, Functional Independence Measure (FIM) motor item scores of < 20 points on activities of daily livings scale and Mann assessment of swallowing ability scores of < 171 points, indicating swallowing function, were reported to be associated with an increased the risk of pneumonia.^[2]^ Based on the above evidence, efforts to reduce the bedridden state and reduce low activity may be necessary to reduce the risk of developing pneumonia in bedridden older patients.

The change of posture from the supine position to sitting upright in a wheelchair is expected to change the ventilation status. In clinical practice, for patients with mobility disorder, maintaining wheelchair sitting and reducing a bedridden state can be important rehabilitation approaches that can help with postural drainage.^[6]^ It has also been previously reported that sitting upright increases functional residual capacity and prevents alveolar collapse.^[7]^ Furthermore, tidal volume (V_T_) and minute volume increased in the sitting posture without changing oxygen uptake (V̇O_2_) compared with the supine posture in individuals who could sit or stand.^[8,9]^ Increasing V_T_ and the expiratory flow rate is useful for sputum expectoration.^[10]^ However, the effects of changes in the wheelchair sitting posture on metabolic and ventilatory status in bedridden older patients are not clear. Furthermore, as many bedridden older people have several comorbidities as well as communication disorders, it is necessary to examine whether the effects of changes in sitting posture on breathing and metabolism are within a safe range.

Therefore, this study aimed to clarify the safety and effects of postural change from the supine position to the reclining position on metabolic and ventilatory parameters in bedridden older patients. By clarifying the effects of postural change in bedridden older patients, we expect to obtain useful information for identifying the optimal physical activity to prevent pneumonia.

## 2. Methods

### 2.1. Ethical consideration

This study was approved by the Ethics Committee of Kofu Kyoritsu Hospital (approval no: 2018-2) and the Ethics Committee of Health Sciences of Kobe University Graduate School of Health Sciences (no: 961). Before the study, explanations about the study were given to the participants and their family members, and written consent was obtained.

### 2.2. Participants

Twenty patients who were hospitalized and treated in an acute care hospital between May and October 2018 participated in this study. The inclusion criteria were as follows: Being older, requiring nursing care, and having an FIM motor score of <20 points; Having cerebrovascular disease as the leading cause of a bedridden state (at least 6 months have passed since the onset of the disease); Not having chronic respiratory failure or chronic heart failure; Not having a fever (temperature <37.5°C) and having a stable medical condition; Having <14 hours of sitting time in a wheelchair per week^[11]^; and; Having rehabilitation prescribed by a physician and having practiced sitting on the edge of a bed for at least 10 minutes with a physical therapist or others, during which the vital signs remained stable.

### 2.3. Measurement procedure

Participants were placed in the following 3 positions: lying on the back (supine), resting in the Fowler position with a 40-degree recline and 20-degree tilt-up (Fowler), and resting in an 80-degree recline wheelchair (80°). Data on respiration and circulation were continuously obtained. The experiment was conducted at a fixed time more than 2 hours after eating for participants who took food orally or enterally. The participants were placed in the supine position for at least 10 minutes before the measurement. After checking heart rate (HR), transcutaneous oxygen saturation (SpO_2_), and blood pressure, the participants wore a face mask and a HR sensor. We confirmed that there was no air leakage when the face mask was put on, and we instructed the participants to breathe spontaneously and not to speak or make any sound during the measurement.

The participants were then seated in a reclining wheelchair in a relaxed position to allow full respiratory function. The trunk was placed in a straight line along the back of the wheelchair, and the lower limbs were placed on a footrest. Measurements were taken with the head and neck in a 30-degree head and neck flexion position (slight flexion) as a pillow was used to stabilize the head and neck.

**Figure 1.**
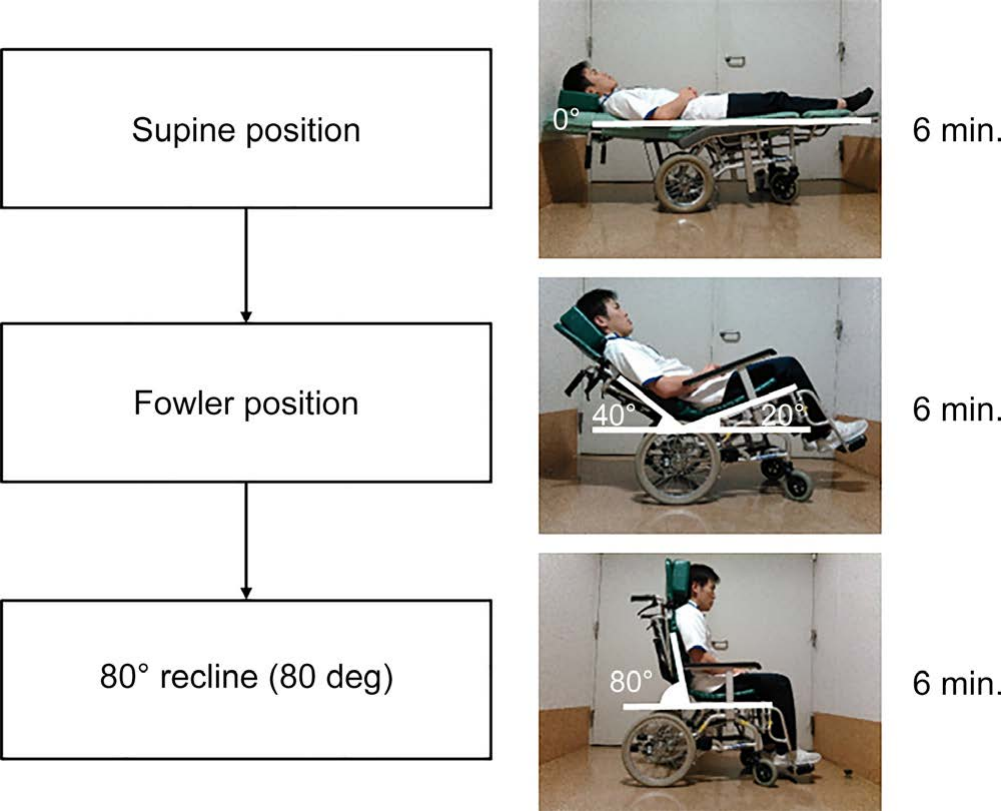
Three postures adopted by the subjects. From top to bottom: supine at rest (supine), 40° reclined and 20° tilt-up (Fowler), and 80° reclined wheelchair (80°). Measurements were taken for 6 minutes in each posture.

Measurements in each position were taken consecutively in the order of supine, Fowler, and 80°. These conditions were set up to approximate those commonly seen in rehabilitation clinical practice, in which patients are intervened in the supine position, then moved to the head-up position, and finally released in the sitting position (Fig. 1).

Measurements were taken for 6 minutes in each position, and data were recorded every 5 seconds.

### 2.4. Apparatus

Exhaled gases were measured by the breath-by-breath method using a breath gas analyzer (Aeromonitor AE-300S; Minato Medical Science Co, Ltd, Osaka, Japan). Calibration of the gas sensor, volume transducer, and barometric pressure sensor was performed before each measurement. Exhaled gases were automatically collected via a face mask and a sample tube. The indices obtained from the expiratory gas measurements were V̇O_2_, V̇O_2_ divided by weight (V̇O_2_/W), carbon dioxide output, gas exchange ratio (R), V_T_, minute volume, respiratory rate, inspiratory time, expiratory time, total respiratory time, mean inspiratory flow (V_T_/Ti), metabolic equivalents, end-expiratory oxygen, and end-expiratory carbon dioxide.

HR and blood pressure were measured using a biometric monitor (WEP4204; Nhon Kohden, Tokyo, Japan). A pulse oximeter (3100 WristOx; Nonin, Minnesota) was used for SpO_2_ and pulse rate.

### 2.5. Participants characteristics

The data on age, gender, body mass index (BMI), name of the disease at admission, medications, FIM scores, Mann assessment of swallowing ability scores, biochemical findings (serum albumin, C-reactive protein, blood urea nitrogen, estimated glomerular filtration rate, and creatinine) were obtained from medical records.

## 3. Statistical analysis

Average values obtained from 3 to 6 minutes (180–360 seconds) in each of the 3 experimental conditions (defined as the steady-state period) were used for analysis. For V̇O_2_ and V_T_, ΔV̇O_2_ (Fowler) and ΔV_T_ (Fowler) were calculated as the change from the supine position to the Fowler position, and ΔV̇O_2_ (80°) and ΔV_T_ (80°) were calculated as the change from the supine position to the 80° position.

The Kolmogorov–Smirnov test was used to analyze the normality of each variable. Multiple comparisons were performed between the 3 positions of supine, Fowler, and 80° using Bonferroni correction. Pearson product-moment correlation coefficient was used to examine the relationship between metabolic and ventilatory parameters (V̇O_2_ and V_T_). In the post hoc power analysis, all variables had a power >0.8. For statistical analysis, EZR (version 1.32) was used for comparisons between positions, and G Power 3.1 (http://www.psycho.uni-duesseldorf.de/abteilungen/aap/gpower3/) software was used for power analysis. The results for continuous variables are presented as mean ± standard deviation, and the results for nominal variables are presented as number of persons (%). Two-tailed *P* *<* .05 was considered statistically significance.

## 4. Results

Of the 20 participants who met the inclusion criteria and participated in the experiment, 19 were included in the analysis after excluding 1 participant who spoke frequently during the experiment. The demographic data of the analyzed participants are shown in Table 1. The mean age of the participants was 83.2 ± 8.4 years, and the mean BMI was 17.3 ± 3.3 kg/m^2^. Of the 19 participants, 12 (63.2%) were male. Moreover, 15 (78.9%) participants had aspiration pneumonia and bacterial pneumonia diseases. The mean serum albumin level was 2.8 ± 0.9 g/dL, which was lower than the reference value (4.1–5.1 g/dL), and the mean C-reactive protein level was 2.1 ± 2.4 mg/dL, which was higher than the reference value (≤0.30 mg/dL).

**Table 1:** Participants characteristics.

Characteristics	All (n = 19)
Age, yr	83.2 ± 8.4
Male (n, %)	12 (63.2)
BMI, kg/m^2^	17.3 ± 3.3
Cause of hospitalization	
Pyelonephritis (n, %)	3 (15.8)
Aspiration pneumonia (n, %)	10 (52.6)
Bacterial pneumonia (n, %)	5 (26.3)
Postdrug-induced bradycardia (n, %)	1 (5.3)
Alb, g/dL	2.8 ± 0.9
BUN, mg/dL	19.2 ± 8.4
Cr, mg/dL	0.7 ± 0.5
CRP, mg/dL	2.1 ± 2.4
eGFR, mL min^−1^1.73m^−2^	98 ± 49.9

Data presented as mean ± SD, or n (%).

BMI = body mass index, alb = albumin, BUN = blood urea nitrogen, Cr = creatinine, CRP = C-reactive protein, eGFR = estimated glomerular filtration rate.

The measured data obtained under each condition and the results of multiple comparisons are shown in Table 2. In the post hoc power analysis, all variables demonstrated a power of > 0.8. There were no significant differences in HR, and SpO2 did not change among the 3 positions. V̇O_2_ increased by 10.8 mL/minute from the supine position (114.6 ± 31.6 mL/minute) to the Fowler position (125.4 ± 27.1 mL/minute) and decreased by 1.4 mL/minute from the Fowler position to the 80° position (124 ± 22.9 mL/minute). No significant difference in V̇O_2_/W was observed among the 3 positions. The R decreased with an increase in the reclining angle from 0.94 ± 0.05 in the supine position to 0.92 ± 0.05 in the 80° position, showing a significantly lower value in the 80° position than in the supine position (*P* *=* .01).

**Table 2:** Changes in measurement parameters due to posture changes.

	Supine	Fowler	80°
HR, bpm	76.0 ± 14.4	75.1 ± 15.0	76.5 ± 15.6
SpO_2_, %	95.5 ± 2.6	95.2 ± 2.1	94.7 ± 2.5
V̇O_2_/W, mL/min/kg	2.9 ± 0.8	3.2 ± 0.5	3.2 ± 0.7
V̇O_2_, mL/min	114.6 ± 31.6	125.4 ± 27.1	124.0 ± 22.9
V̇CO_2_, mL/min	107.6 ± 31.6	116.6 ± 27.9	114.3 ± 24.9
R	0.94 ± 0.1	0.93 ± 0.1	0.92 ± 0.1 †
V_T_, mL	398.4 ± 112.2	426.9 ± 106.8 *	416.8 ± 92.5
VE, L/min	6.5 ± 1.4	7.1 ± 0.6	7.2 ± 1.5
RR, bpm	16.8 ± 3.0	17.0 ± 3.3	17.7 ± 3.8
Ti, s	1.4 ± 0.3	1.4 ± 0.3	1.4 ± 0.4
Te, s	2.4 ± 0.5	2.3 ± 0.5	2.3 ± 0.7
Ttot, s	3.8 ± 0.7	3.7 ± 0.7	3.7 ± 0.3
VT/Ti, mL/s	281.1 ± 54.1	307.6 ± 59.2 *	314.8 ± 75.7

The results for continuous variables are presented as mean ± standard deviation.

HR = heart rate, R = gas exchange ratio, RR = respiratory rate, SpO2 = transcutaneous oxygen saturation, Te = expiratory time, Ti = inspiratory time, Ttot = total respiratory time, V̇CO_2_ = oxygen uptake, VE = minute volume, V̇O2 = carbon dioxide output, V̇O2/W = oxygen uptake divided by weight, VT = tidal volume, VT/Ti = mean inspiratory flow.

* Bonferroni correction was used to compare between the supine and Fowler position.

† Bonferroni correction was used to compare between the supine and 80° position.

V_T_ significantly increased from the supine position (398.4 ± 111.2 mL) to the Fowler position (426.9 ± 106.8 mL) (*P* *=* .037) and showed a decreasing trend from the Fowler position to the 80° position (416.8 ± 92.5 mL). The respiratory rate and minute ventilation rate tended to increase in both the Fowler and 80° positions, whereas inspiratory time and total respiratory time tended to decrease with postural change. V_T_/Ti was significantly higher in the supine and Fowler positions than in the 80° position (*P* *=* .02).

**Figure 2.**
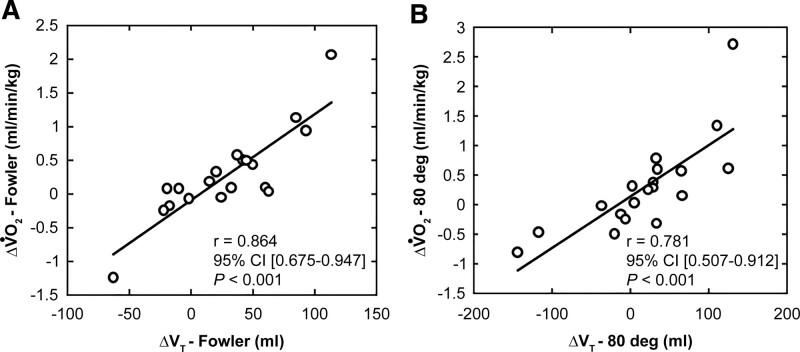
Scatter plots of the change in oxygen uptake (V̇O_2_) and tidal volume (V_T_) caused by the change from the supine position. A) from supine to Fowler and B) from supine to 80°. The black lines represent regression lines. Pearson product-moment correlation coefficient was used to examine the relationship between metabolic and ventilatory parameters (V̇O_2_ and V_T_).

We analyzed the relationship between the changes in V_T_ and V̇O_2_ by changing the posture from supine to Fowler and 80°. Figure 2A presents a scatter plot of ΔV̇O_2_ (Fowler) and ΔV_T_ (Fowler) calculated as the change from the supine position to the Fowler position. Figure 2B presents a scatter plot of ΔV̇O_2_ (80°) and ΔV_T_ (80°) calculated as the change from the supine position to the 80° position. A significant positive correlation was found between ΔV̇O_2_ (Fowler) and ΔV_T_ (Fowler) (*R* = 0.864, 95% confidence interval [0.675 − 0.947], *P ˂*.001). Similarly, there was a significant positive correlation between ΔV̇O_2_ (80°) and ΔV_T_ (80°) (*R* = 0.781, 95% confidence interval [0.507–0.912], *P ˂*.001).

## 5. Discussion

The purpose of the present study was to clarify the effects of postural change from the supine to reclining position on metabolic and ventilatory parameters in bedridden older patients. The following 3 findings were obtained in this study: For bedridden older patients, the change in V̇O_2_ driven by changing the posture from the supine position to the Fowler position was as small as 10.8 mL/minute, which corresponds to an extremely low-intensity physical activity; The ventilatory volume slightly increased from the supine position to the Fowler position, and; although the increase in ΔV̇O_2_ by changing posture was small, an increase in ΔV_T_ was found, and there was a significant positive correlation between ΔV̇O_2_ (Fowler) and ΔV_T_(Fowler), and between ΔV̇O_2_ (80°) and ΔV_T_ (80°). These results indicated that elevating the trunk from the supine position in a bed enhances oxygen uptake as well as the volume of single ventilation.

Wheelchair seating is a very low-impact physical activity for bedridden older patients as well as for healthy people. In a study that set 70-degree tilt-up sitting for healthy participants, V̇O_2_ increased by 10 mL/minute from the supine position.9 In a study with acute stroke patients, there was a 10 mL/minute decrease in V̇O_2_ with a 30-degree head-up from the supine position, a 10 mL/minute increase in the sitting position, and a 140 mL/minute increase in the standing position, indicating that the physical load is high in the standing position.^[12]^ In the present study, the change in V̇O_2_ was found to be 10.8 mL/minute, an increase similar to that in healthy people, when sitting upright during the acute phase of stroke. This increase was small and was not considered as a clinically meaningful change.

V̇O_2_ increases with increasing exercise load, and its determinants are cardiac output, HR, and arteriovenous oxygenation rate.^[13]^ In the reclining wheelchair posture used in the present study, the increase in V̇O_2_, an index of metabolic rate, may have been small because the reclining wheelchair posture does not require the activity of antigravity muscles and the oxygen demand of skeletal muscles is not increased. Furthermore, the wheelchair seating positions used in this experiment (the Fowler position and 80° position) did not cause significant changes in the participants circulatory dynamics, as the changes in HR and SpO_2_ were minimal. Therefore, for older bedridden patients, sitting in a wheelchair is indicated as a very low-intensity, safe physical activity demonstrating no remarkable changes in vital signs.

V_T_ in the bedridden older patients was maximal in the Fowler position and differed from that in normal people. During postural change from the supine position to the Fowler position, V_T_ increased by 28.5 mL (*P* = .037) and the V_T_/Ti increased by 26.5 mL/s (*P* = .02). Both values were significantly higher than the supine position values. This is consistent with the findings in a previous study that showed an increase in lung volume at 45° and 60° bed angles in patients with ARDS.^[14]^ The V_T_/Ti (mL/s) is also recognized as an indicator of ventilatory drive,^[15]^ and it increases with increasing exercise load.^[16]^ Furthermore, the correlation analysis showed a significant positive correlation between ΔV̇O_2_ and ΔV_T_, suggesting that postural change to the reclining sitting position can increase V_T_, a ventilation index, and promote ventilatory drive, even though it is a low-intensity activity for bedridden older patients. However, in normal people, it has been reported that ventilation increases as the backrest tilt increases from the supine position.^[9,17]^ In the present study, V_T_ was maximal in the Fowler position and was mildly decreased in the 80° position, indicating that V_T_ in bedridden older patients is different from that in normal people.

Ventilation did not increase with an increase in the reclining angle. The reason for this result may be the background specific to bedridden older patients. In general, chest wall motion in the sitting posture is associated with less gravitational compression of the thorax, higher thoracic compliance, mechanically favorable intercostal muscle length and contraction, and lower resistance to diaphragmatic contraction than in the supine position, resulting in increased ventilation compared to that in the supine and Fowler positions.^[18]^ In contrast, in older people, age-related structural changes, such as round back and decreased mobility of the thorax, are thought to affect respiratory function.^[19]^ In addition, respiratory function is adapted to a narrow range of physical activity states, and it stabilizes at a low level in bedridden cases.^[20]^ Therefore, among the bedridden older participants in the present study, anterior tilt and a circular back posture with an increasing reclining angle made antigravity extension of the trunk difficult, and limited thoracic movement in the direction of gravity^[21]^ may have caused the decrease in ventilation volume in the 80° position. Further evaluation of the setting of the reclined sitting angle for bedridden older patients in clinical situations is needed.

This study has several limitations. First, since this was a single-center study and the number of participants was not large, we need to be cautious in generalizing the results of this study. Second, the degree of round back was not measured. It is necessary to clarify in a future study how much dorsal circularity affects the ventilation rate in the 80° position. Third, the bedridden older participants in this study were assumed to be in a stable condition after recovery from a condition that required hospitalization. Therefore, metabolic and ventilatory indices were evaluated after recovery. However, we could not exclude the influence of the causative disease at the time of admission. In addition, many of the participants had a poor nutritional condition. This may have decreased the muscle mass of the whole body, including respiratory muscles, and may have affected the results of metabolic and ventilatory indices. In order to further analyze postural and ventilatory changes in bedridden older patients, it is necessary to recruit participants who live at home or in facilities, have confirmed clinical stability, and have a good nutritional condition.

The results of this study indicated that V_T_, an index of respiratory function, changes with postural manipulation. Moreover, we showed that respiratory function (VC, FVC, and PImax) is associated with peak cough flow (PCF),^[22,23]^ which is used to measure the coughing capacity, and low PCF is associated with the development of pneumonia.^[24]^ Several bedridden older patients, similar to those included in this study, have difficulty in communicating, and measuring their VC, FVC, and PCF is often difficult. Future analyses of the relationship among V_T_, PCF, and pneumonia in the appropriate reclining wheelchair sitting position may yield the development of programs that can help prevent pneumonia in bedridden older patients.

We demonstrated that among bedridden older patients, sitting in a wheelchair is a safe and very low-intensity physical activity. V_T_ was maximal in the Fowler position, and the ventilatory volume did not increase with an increase in the reclining angle. These findings suggest that appropriate reclining postures in clinical situations can promote an increase in the ventilatory rate in bedridden older patients.

## Acknowledgements

We are indebted to patients who agreed to participate and cooperate in this study. The authors would like to thank the members of the Department of Rehabilitation, Kofu Kyoritsu Hospital, including Nana Muranaka, Ryota Sakai, Genki Komatsu, and Taiki Miura, for their cooperation in the acquisition of data. Finally, the authors would like to thank the Kobe University Graduate School of Health Sciences, including Kaoru Hanaie, Takumi Yamaguchi, Shigehumi Murakami, Kentaro Iwata, Yusuke Iwata, Mariko Fujita, Ken Umehara, Keita Ohashi, Haruko Suzuki, Kohei Otake, Hiroki Mizusawa, Masaaki Kobayashi, for their constructive comments on this paper. We would like to thank Enago for English language editing.

## Author contributions

**Conceptualization:** Yuji Mitani, Akio Yamamoto, Kazumo Miura, Kanji Yamada, Yukari Oki, Yutaro Oki, Yoko Kurumatani, Akira Ishikawa.

**Data curation:** Yoji Yamada, Yasumichi Maejima.

**Formal analysis:** Yoji Yamada, Akio Yamamoto.

**Writing – original draft:** Yoji Yamada, Akio Yamamoto.

**Writing – review & editing:** Yoji Yamada, Akio Yamamoto, Kanji Yamada, Yutaro Oki, Akira Ishikawa.
